# Dynamic transfer learning with progressive meta-task scheduler

**DOI:** 10.3389/fdata.2022.1052972

**Published:** 2022-11-03

**Authors:** Jun Wu, Jingrui He

**Affiliations:** ^1^Department of Computer Science, University of Illinois at Urbana-Champaign, Champaign, IL, United States; ^2^School of Information Sciences, University of Illinois at Urbana-Champaign, Champaign, IL, United States

**Keywords:** transfer learning, distribution shift, dynamic environment, meta-learning, task scheduler, image classification

## Abstract

Dynamic transfer learning refers to the knowledge transfer from a static source task with adequate label information to a dynamic target task with little or no label information. However, most existing theoretical studies and practical algorithms of dynamic transfer learning assume that the target task is continuously evolving over time. This strong assumption is often violated in real world applications, e.g., the target distribution is suddenly changing at some time stamp. To solve this problem, in this paper, we propose a novel meta-learning framework L2S based on a progressive meta-task scheduler for dynamic transfer learning. The crucial idea of L2S is to incrementally learn to schedule the meta-pairs of tasks and then learn the optimal model initialization from those meta-pairs of tasks for fast adaptation to the newest target task. The effectiveness of our L2S framework is verified both theoretically and empirically.

## 1. Introduction

**Figure 1:**
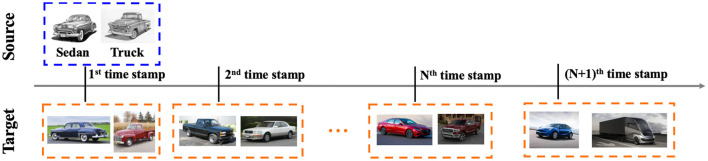
Illustration of dynamic transfer learning from a static source task (e.g., sketch image classification with fully labeled examples) to a dynamic target task (e.g., real-world image classification with only unlabeled examples).

Transfer learning (Pan and Yang, 2009; Tripuraneni et al., 2020) improves the generalization performance of a learning algorithm on the target task, by leveraging the knowledge from a relevant source task. It has been studied (Ben-David et al., 2010; Long et al., 2015; Ganin et al., 2016; Zhang et al., 2019) that the knowledge transferability across tasks can be theoretically guaranteed under mild conditions, e.g., source and target tasks share the same labeling function. One assumption behind those works is that source and target tasks are sampled from a stationary task distribution. More recently, it is observed that in the context of transfer learning, the tasks might be sampled from a non-stationary task distribution, i.e., the learning task might be evolving over time in real scenarios. It can be formulated as a dynamic transfer learning problem from a static source task^1^ with adequate label information to a dynamic target task with little or no label information (see Figure 1).

Most existing works (Hoffman et al., 2014; Bobu et al., 2018; Kumar et al., 2020; Wang H. et al., 2020; Wu and He, 2020, 2022b) on dynamic transfer learning assume that the target task is continuously changing over time. This assumption allows deriving the generalization error bound of dynamic transfer learning using the distribution shift at any consecutive time stamps. Nevertheless, we show that these error bounds are not tight when the task distribution changes suddenly at some time stamp. Therefore, previous works can be hardly applied to real scenarios where the task distribution might not always be evolving continuously. This sudden distribution shift can be induced by some unexpected issues, e.g., adversarial attacks (Wu and He, 2021), system failures (Lu et al., 2018), etc.

To solve this problem, we derive the generalization error bound of dynamic transfer learning in terms of adaptively scheduled meta-pairs of tasks. Moreover, it is observed that this result is closely related to the existing error bounds (Wang et al., 2022; Wu and He, 2022b). It is found that previous works showed the error bounds in terms of the distribution shift at any consecutive time stamps. In contrast, we consider all the meta-pairs of tasks, e.g., a pair of tasks transferring the knowledge from an old time stamp to a new time stamp. As a result, our error bound can be tight even when the task distribution is suddenly shifted at some time stamp. Then, by minimizing the error bound, we propose a novel meta-learning framework L2S based on a progressive meta-task scheduler for dynamic transfer learning. In this framework, we automatically learn the sampling probability for meta-pairs of tasks based on task relatedness. The effectiveness of L2S framework is then verified on a variety of dynamic transfer learning tasks. The major contributions of this paper are summarized as follows.

- We consider a relaxed assumption of dynamic transfer learning, i.e., the target task distribution might change suddenly at some time stamp when it is evolving over time. The generalization error bounds of dynamic transfer learning can then be derived with this relaxed assumption.
- We propose a novel meta-learning framework L2S based on a progressive meta-task scheduler for dynamic transfer learning. Different from recent work (Wu and He, 2022b), L2S learns to schedule the meta-pairs of tasks based on task relatedness.
- Experiments on various data sets demonstrate the effectiveness of our L2S framework over state-of-the-art baselines.

The rest of the paper is organized as follows. We review the related work in Section 2. The problem of dynamic transfer learning is defined in Section 3. In Section 4, we derive the error bounds of dynamic transfer learning, followed by the proposed L2S framework in Section 5. The empirical analysis on L2S is provided in Section 6. Finally, we conclude the paper in Section 7.

## 2. Related work

In this section, we briefly introduce the related work on dynamic transfer learning and meta-learning.

### 2.1. Dynamic transfer learning

Dynamic transfer learning (Hoffman et al., 2014; Bitarafan et al., 2016; Mancini et al., 2019) refers to the knowledge transfer from a static source task to a dynamic target task. Compared to standard transfer learning on the static source and target tasks (Pan and Yang, 2009; Zhou et al., 2017, 2019a,b; Tripuraneni et al., 2020; Wu and He, 2021), dynamic transfer learning is a more challenging but realistic problem setting due to its time evolving task relatedness. More recently, various dynamic transfer learning frameworks are built from the following aspects: self-training (Kumar et al., 2020; Chen and Chao, 2021; Wang et al., 2022), incremental distribution alignment (Bobu et al., 2018; Wulfmeier et al., 2018; Wang H. et al., 2020; Wu and He, 2020, 2022a), meta-learning (Liu et al., 2020; Wu and He, 2022b), contrastive learning (Tang et al., 2021; Taufique et al., 2022), etc. Specifically, most existing works assume that the task distribution is continuously evolving over time. Very little effort has been devoted to studying dynamic transfer learning when this assumption is violated in real scenarios. Compared to previous works (Liu et al., 2020; Wang et al., 2022; Wu and He, 2022b), in this paper, we focus on a more realistic dynamic transfer learning with a relaxed assumption that the task distribution could be suddenly changed at some time stamp.

### 2.2. Meta-learning

Meta-learning (Hospedales et al., 2021) leverages the knowledge from a set of prior meta-training tasks for fast adaptation to new tasks. In the context of few-shot classification, meta-learning aims to find the optimal model initialization (Finn et al., 2017, 2018; Wang L. et al., 2020; Yao et al., 2021) from previously seen tasks such that this model can be fine-tuned on a new task by performing a few gradient steps. It assumes that all the tasks follow a stationary task distribution. More recently, this meta-learning paradigm has been extended into the online learning setting where a sequence of tasks is sampled from non-stationary task distributions (Finn et al., 2019; Acar et al., 2021). Following previous work (Wu and He, 2022b), we formulate dynamic transfer learning as a meta-learning problem, which aims to learn the optimal model initialization for knowledge transfer across any meta-pair of tasks. In contrast to Wu and He (2022b) where the meta-pairs of tasks are simply constructed from tasks at consecutive time stamps, we propose to learn the sampling probability for meta-pairs of tasks based on the task relatedness during model training. This can help our meta-learning framework avoid the negative transfer induced by the meta-pairs of tasks sampled from suddenly shifted task distribution.

## 3. Preliminaries

In this section, we present the notation and formal problem definition of dynamic transfer learning.

### 3.1. Notation

Let X and Y be the input feature space and output label space respectively. We consider the dynamic transfer learning problem (Hoffman et al., 2014; Bobu et al., 2018) with a static source task $D^{s}$ and a dynamic target task ${\left\{D_{j}^{t}\right\}}_{j=1}^{N}$ with time stamp *j*. In this case, we assume that there are *m*^*s*^ labeled training examples $D^{s}={\left\{\left({\text{x}}_{i}^{s},y_{i}^{s}\right)\right\}}_{i=1}^{m^{s}}$ in the source task. Let $m_{j}^{t}$ be the number of unlabeled training examples $D_{j}^{t}={\left\{{\text{x}}_{ij}^{t}\right\}}_{i=1}^{m_{j}^{t}}$ in the *j*^th^ target task. Let H be the hypothesis class on X where a hypothesis is a function h:X→Y. L(·,·) is the loss function such that L:Y×Y→ℝ. The expected classification error on the source task $D^{s}$ is defined as $\epsilon^{s}\left(h\right)=E_{\left(\text{x},y\right)\sim D^{s}}\left[L\left(h\left(\text{x}\right),y\right)\right]$ for any h∈H, and its empirical estimate is given by ${\hat{\epsilon }}^{s}\left(h\right)=\frac{1}{m^{s}}\overset{m^{s}}{\underset{i=1}{\sum }}L\left(h\left({\text{x}}_{i}\right),y_{i}\right)$. The expected error $\epsilon_{j}^{t}\left(h\right)$ and empirical error ${\hat{\epsilon }}_{j}^{t}\left(h\right)$ of the target task at the *j*^th^ time stamp can also be defined similarly.

### 3.2. Problem definition

Following previous works (Hoffman et al., 2014; Bitarafan et al., 2016; Bobu et al., 2018), we formally define the problem of dynamic transfer learning as follows.

** Definition 3.1**. *(Dynamic Transfer Learning) Given a labeled static source task $D^{s}$ and an unlabeled dynamic target task ${\left\{D_{j}^{t}\right\}}_{j=1}^{N}$, dynamic transfer learning aims to learn the prediction function for the newest target task $D_{N+1}^{t}$ by leveraging the knowledge from historical source and target tasks*.

**Figure 2:**
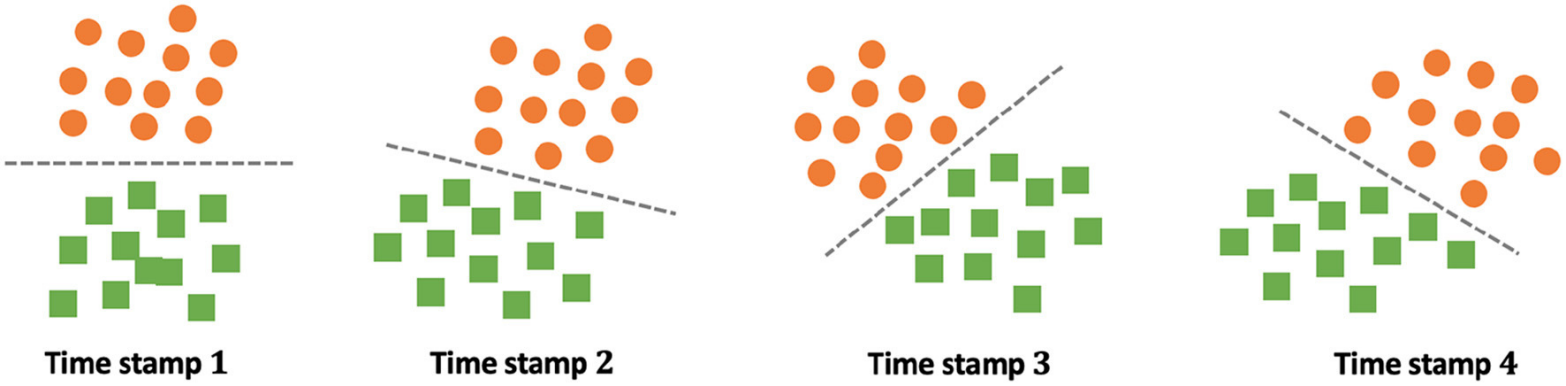
Challenges of dynamic transfer learning where the task distribution is suddenly changed at time stamp 3. Here orange circle and green square denote data points from two classes, and the dashed line indicates the optimal decision boundary at different time stamps.

The key challenge of dynamic transfer learning is the time evolving task relatedness between source and target tasks. Recent works (Liu et al., 2020; Wang et al., 2022; Wu and He, 2022b) showed the generalization error bounds by assuming that the data distribution of the target task is continuously changing over time. Intuitively, in this case, the expected error bound on the newest target task is bounded in terms of the largest distribution gap [e.g., $\underset{0\leq j\leq N}{\max}d_{H\Delta H}\left(D_{j}^{t},D_{j+1}^{t}\right)$] across time stamps. As a result, these generalization error bounds are not tight when the task distribution is significantly shifted at some time stamp. As shown in Figure 2, the task distribution is shifted smoothly from time stamp 1 to time stamp 2. However, it changes sharply from time stamp 2 to time stamp 3. In real scenarios, this sharp distribution shift might be induced by some unexpected issues, e.g., adversarial manipulation (Wu and He, 2021). This thus motivates us to study dynamic transfer learning with a much more relaxed assumption that the task distribution could be suddenly shifted at some time stamp.

## 4. Theoretical analysis

In this section, we provide the theoretical analysis for dynamic transfer learning.

### 4.1. Generalization error bound

We derive the generalization error bound of dynamic transfer learning as follows. Following Ben-David et al. (2010) and Liu et al. (2020), we use H-divergence to measure the distribution shift across tasks and Vapnik-Chervonenkis (VC) dimension to measure the complexity of a class of functions H. Without loss of generality, we would like to consider a binary classification problem (i.e., Y={0,1}) with the loss function L(ŷ,y)=|ŷ-y|. The following theorem showed that the expected error of the newest target task $D_{N+1}^{t}$ can be bounded in terms of the historical source and target knowledge.

** Theorem 4.1**. *(Generalization Error Bound) Let H be a hypothesis space of VC dimension *d*. If there are *m* labeled source examples i.i.d. drawn from $D^{s}$ (denoted as $D_{0}^{t}$ as well) and *m* unlabeled target examples i.i.d. drawn from $D_{j}^{t}$ for each time stamp *j* = 1, ⋯ , *N*+1^2^, then for any δ>0 and h∈H, with probability at least 1−δ, the expected error of the newest target task $D_{N+1}^{t}$ can be bounded as follows*.


$$\begin{array}{l} \epsilon_{N+1}^{t}(h)\leq \sum_{i=0}^{N}\sum_{j=i+1}^{N+1}w_{ij}({\hat{\epsilon }}_{i}^{t}(h)+\eta_{ij}\cdot {\hat{d}}_{ℋ\Delta ℋ}\left(D_{i}^{t},D_{j}^{t}\right))\;\\ +O\left(\lambda +\sqrt{\frac{d\log(2m)+\log(2/\delta )+\sum_{i=0}^{N}\sum_{j=i+1}^{N+1}w_{ij}^{2}\log(1/\delta )}{2m}}\right) \end{array}$$

*where $\overset{N}{\underset{i=0}{\sum }}\overset{N+1}{\underset{j=i+1}{\sum }}w_{ij}=1$, and *w*_*ij*_≥0 if *i*<*j*, *w*_*ij*_ = 0 otherwise. $\eta_{ij}=\frac{1}{2}$ if 1 ≤ *j* ≤ *N* and *i*<*j*, and $\eta_{ij}=\frac{1}{2}\left(1+\frac{\overset{i-1}{\underset{k=0}{\sum }}w_{ki}}{w_{ij}}\right)$ if *j* = *N*+1 and *i*<*j*, η_*ij*_ = 0 otherwise. Here λ denotes the combined error of the ideal hypothesis over all the tasks, i.e., $\lambda =\underset{h\in H}{\min}\overset{N+1}{\underset{i=0}{\sum }}\epsilon_{i}^{t}\left(h\right)$, and ${\hat{d}}_{H\Delta H}\left(\cdot ,\cdot \right)$ denotes the empirical estimate of H-divergence over finite examples*.

Note that this error bound holds with other existing distribution discrepancy measures (see Corollary 4.3), though we consider H-divergence (Ben-David et al., 2010) in Theorem 4.1. Furthermore, we show the generalization error bound of dynamic transfer learning from the perspective of meta-learning. That is, instead of sharing the hypothesis h∈H for all the tasks, we learn a common initialized model $\bar{h}\in H$ across tasks. Then the task-specific model *h*_*i*_ via one-step gradient update for the target at the *i*^th^ time stamp, i.e., $\theta_{i}=\bar{\theta }-\beta \nabla_{\theta }L^{meta}$, where $\theta_{i},\bar{\theta }$ denotes the parameters of $h_{i},\bar{h}$ respectively and $L^{meta}$ is the meta-learning loss for updating the task-specific model parameters. If we let $L^{meta}={\hat{\epsilon }}_{i}^{t}\left(\bar{h}\right)=\frac{1}{m}\overset{m}{\underset{k=1}{\sum }}L\left[\bar{h}\left({\text{x}}_{ki}\right),y_{ki}\right]$, the following theorem provides the generalization error bound based on meta-learning.

** Theorem 4.2**. *(Meta-Learning Generalization Error Bound) Let H be a hypothesis space of VC dimension *d*. If there are *m* labeled source examples i.i.d. drawn from $D^{s}$ (denoted as $D_{0}^{t}$ as well) and *m* unlabeled target examples i.i.d. drawn from $D_{j}^{t}$ for each time stamp *j* = 1, ⋯ , *N*+1, then for any δ>0 and a proper inner learning rate β, with probability at least 1−δ, the expected error of the newest target task $D_{N+1}^{t}$ can be bounded in the following*.


$$\begin{array}{l} \epsilon_{N+1}^{t}(h_{N+1})\leq \sum_{i=0}^{N}\sum_{j=i+1}^{N+1}w_{ij}({\hat{\epsilon }}_{i}^{t}(h_{i})+\eta_{ij}\cdot {\hat{d}}_{ℋ\Delta ℋ}\left(D_{i}^{t},D_{j}^{t}\right))\\ +O(\sum_{i=0}^{N}{\left(\frac{1}{m}\sum_{k=1}^{m}\left|\left|\nabla_{\theta }\bar{h}(x_{ki})\right|\right|\right)}^{2}\\ +\lambda +\sqrt{\frac{d\log(2m)+\log(2/\delta )+\sum_{i=0}^{N}\sum_{j=i+1}^{N+1}w_{ij}^{2}\log(1/\delta )}{m}}) \end{array}$$

*where $\overset{N}{\underset{i=0}{\sum }}\overset{N+1}{\underset{j=i+1}{\sum }}w_{ij}=1$, and *w*_*ij*_≥0 if *i*<*j*, *w*_*ij*_ = 0 otherwise. $\eta_{ij}=\frac{1}{2}$ if 1 ≤ *j* ≤ *N* and *i*<*j*, and $\eta_{ij}=\frac{1}{2}\left(1+\frac{\overset{i-1}{\underset{k=0}{\sum }}w_{ki}}{w_{ij}}\right)$ if *j* = *N*+1 and *i*<*j*, η_*ij*_ = 0 otherwise. Here λ denotes the combined error of the ideal hypothesis over all the tasks, i.e., $\lambda =\underset{h\in H}{\min}\overset{N+1}{\underset{i=0}{\sum }}\epsilon_{i}^{t}\left(h\right)$, and ${\hat{d}}_{H\Delta H}\left(\cdot ,\cdot \right)$ denotes the empirical estimate of H-divergence over finite examples*.

We observe from Theorem 4.2 that the parameter *w*_*ij*_ plays an important role in the generalization error bound of dynamic transfer learning. Intuitively, it is more likely to assign higher value *w*_*ij*_ for the easy meta-pair of tasks $D_{i}\to D_{j}$ with stronger class discrimination over $D_{i}$ [i.e., smaller ${\hat{\epsilon }}_{i}^{t}\left(h_{i}\right)$] and smaller distribution shift between $D_{i}$ and $D_{j}$ [i.e., smaller ${\hat{d}}_{H\Delta H}\left(D_{i}^{t},D_{j}^{t}\right)$].

### 4.2. Connection to existing bounds

The following corollary shows that the error bound in Theorem 4.1 can be generalized by considering various domain discrepancy measures.

** Corollary 4.3**. *With the same assumptions in Theorem 4.1, for any δ>0 and h∈H, there exist *w*_*ij*_≥0 and η_*ij*_≥0, with probability at least 1−δ, the expected error of the newest target task $D_{N+1}^{t}$ can be bounded in the following*.


(1) $$\begin{array}{l} \epsilon_{N+1}^{t}\left(h\right)\leq \overset{N}{\underset{i=0}{\sum }}\overset{N+1}{\underset{j=i+1}{\sum }}w_{ij}\left({\hat{\epsilon }}_{i}^{t}\left(h\right)+\eta_{ij}\cdot \hat{d}\left(D_{i}^{t},D_{j}^{t}\right)\right)+\Omega \end{array}$$

*where $\hat{d}\left(\cdot ,\cdot \right)$ can be instantiated with existing distribution discrepancy measures, including discrepancy distance (Mansour et al., 2009), maximum mean discrepancy (Long et al., 2015), Wasserstein distance (Shen et al., 2018), *f*-divergence (Acuna et al., 2021), etc. Here Ω denotes the corresponding sample complexity when the distribution discrepancy measure is selected*.

Corollary 4.3 shows the flexibility in generalizing existing static transfer learning theories (Mansour et al., 2009; Ben-David et al., 2010; Ghifary et al., 2016; Shen et al., 2018; Zhang et al., 2019; Acuna et al., 2021) into the dynamic transfer learning setting. Moreover, it is observed that Corollary 4.3 is closely related to the existing generalization error bounds (Wang et al., 2022; Wu and He, 2022b) of dynamic transfer learning, under different parameters *w*_*ij*_ and η_*ij*_.

- When *w*_*ij*_ and η_*ij*_ are given by

$$w_{ij}=\{\begin{array}{ll} \frac{1}{N+1}, & \text{if }i=0\\ \frac{\tau }{N+1}, & \text{if }1\leq i\leq N\text{ and }i+1=j\\ 0, & \text{otherwise} \end{array}$$

$$\eta_{ij}=\{\begin{array}{ll} \rho \sqrt{R^{2}+1}(N+1), & \text{if }i=0\text{ and }j=1\\ \rho \sqrt{R^{2}+1}(N+1)/\tau , & \text{if }1\leq i\leq N\text{ and }i+1=j\\ 0, & \text{otherwise} \end{array}$$

where τ∈ℝ. Then, when τ → 0, Corollary 4.3 recovers the generalization error bound (Wang et al., 2022).


$$\begin{array}{l} \epsilon_{N+1}^{t}(h_{N+1})\leq \epsilon^{s}(h_{0})+\rho \sqrt{R^{2}+1}\sum_{i=1}^{N+1}d_{W_{p}}\left(D_{i-1}^{t},D_{i}^{t}\right)\\ +O(N\sqrt{\frac{\log(1/\delta )}{m}}+\frac{N}{\sqrt{m}}+\frac{1}{\sqrt{mN}}+\sqrt{\frac{\log{\left(mN\right)}^{3L-2}}{mN}}\\ +\sqrt{\frac{\log(1/\delta )}{mN}}) \end{array}$$

where H is the hypothesis class of *R*-Lipschitz *L*-layer fully-connected neural networks with 1-Lipschitz activation function.

- When *w*_*ij*_ and η_*ij*_ are given by

$$w_{ij}=\{\begin{array}{ll} \frac{1}{N+1}, & \text{if }i+1=j\\ 0, & \text{otherwise} \end{array}\;\eta_{ij}=\{\begin{array}{ll} 1, & \text{if }i+1=j\\ 0, & \text{otherwise} \end{array}$$

Then, Corollary 4.3 recovers the generalization error bound (Wu and He, 2022b).


(2) $$\begin{array}{l} \epsilon_{N+1}^{t}(h)\leq \sum_{i=1}^{N+1}\frac{1}{N+1}\left({\hat{\epsilon }}_{i-1}^{t}(h)+{\hat{d}}_{ℋ\Delta ℋ}\left(D_{i-1}^{t},D_{i}^{t}\right)\right)\\ \;+\Omega_{L} \end{array}$$

where Ω_*L*_ is a Rademacher complexity term.

Compared to existing theoretical results (Wang et al., 2022; Wu and He, 2022b), with appropriate *w*_*ij*_, our generalization error bound in Corollary 4.3 is much more tighter when there exists some time stamp *i* such that ${\hat{d}}_{H\Delta H}\left(D_{i-1}^{t},D_{i}^{t}\right)$ is large. It thus motivates us to develop a progressive meta-task scheduler in the meta-learning framework for dynamic transfer learning. The crucial idea is to automatically learn the values *w*_*ij*_, based on the intuition that assigning large value *w*_*ij*_ on easy meta-pair of tasks $D_{i}\to D_{j}$ would make our error bound much tighter.

## 5. Methodology

Following Wu and He (2022b), we propose a meta-learning framework named L2S for dynamic transfer learning by empirically minimizing the error bound in Theorem 4.2. Instead of uniformly sampling the meta-pairs of tasks in the consecutive time stamps (Wu and He, 2022b), in this paper, we learn a progressive meta-task scheduler for automatically formulating the meta-pairs of tasks from the dynamic target task.

The overall objective function of L2S for learning the prediction function of $D_{N+1}^{t}$ on the (*N*+1)^th^ time stamp is given as follows.


(3) minθ minw J(θ,w)=∑i=0N∑j=i+1N+1wij(ϵ^it(Mij(θ))+η·d^ℋΔℋ(Dit,Djt;Mij(θ)))s.t.   ∑i=0N∑j=i+1N+1wij=1 s.t.   Mij(θ)=θ−β∇θℒmeta(Dit,Djt)

where θ is the trainable parameters and $L^{meta}\left(D_{i}^{t},D_{j}^{t}\right)$ is the meta-training loss. η≥0 is a hyper-parameter to balance the classification error and discrepancy minimization.

The proposed L2S framework has three crucial components: meta-pairs of tasks, meta-training, and meta-testing. The overall training procedures of L2S are illustrated in Algorithm 1.

- **Meta-Pairs of Tasks:** Following the theoretical results in Section 4.1, we formulate the candidate meta-pairs of tasks from any two different time stamps $\left(D_{i}^{t},D_{j}^{t}\right)$ (*i*<*j*). It can be considered as a simple knowledge transfer from $D_{i}^{t}$ to $D_{j}^{t}$. Here we simply denote the source task $D^{s}$ as $D_{0}^{t}$. Since we focus on learning the prediction function on the target task at a new time stamp, we consider the knowledge transfer from an old time stamp *i* to a new time stamp *j*, i.e., *i*<*j*. Note that as suggested in Theorem 4.2, those candidate meta-pairs of tasks might not have equal sampling probability for meta-training. Therefore, we propose a progressive meta-pair scheduler to incrementally learn the sampling probability of every candidate meta-pair of tasks.

As shown in Theorem 4.2, the sampling probability *w*_*ij*_ is strongly related to the classification error on $D_{i}^{t}$ and the empirical distribution discrepancy between $D_{i}^{t}$ and $D_{j}^{t}$. However, we have only unlabeled training examples for the target task. It is intractable to accurately estimate the classification error on $D_{i}^{t}$ (*i* = 1, 2, ⋯ ) for the target task. One solution is that we can incrementally estimate the pseudo-labels of unlabeled target examples, and then obtain the classification error using these pseudo-labels. But it will be largely affected by the quality of the pseudo-labels. Instead, in this paper, we simply learn the sampling probability using the empirical distribution discrepancy between $D_{i}^{t}$ and $D_{j}^{t}$ because this distribution discrepancy involves only the unlabeled examples. That is, the sampling probability *w*_*ij*_ is learned as follows.

(4) $$\begin{array}{l} w_{ij}=\frac{\exp\left(1/{\hat{d}}_{H\Delta H}\left(D_{i}^{t},D_{j}^{t}\right)\right)}{\Gamma } \end{array}$$

where Γ is a normalization term. it indicates that the meta-pair of tasks with a smaller distribution discrepancy has a larger probability of being sampled for meta-training. Intuitively, the smaller distribution discrepancy guarantees the knowledge transfer across tasks (Ganin et al., 2016; Zhang et al., 2019). Therefore, we can sample a set of meta-pairs of tasks S based on the sampling probability for meta-training.

- **Meta-Training:** Following Wu and He (2022b), the meta-training over meta-pairs of tasks is given as follows. Let $\zeta_{ij}\left(\theta \right)={\hat{\epsilon }}_{i}^{t}\left(M_{ij}\left(\theta \right)\right)+\eta \cdot {\hat{d}}_{H\Delta H}\left(D_{i}^{t},D_{j}^{t};M_{ij}\left(\theta \right)\right)$ be the loss function over the validation set on a meta-pair of tasks. Then the model initialization θ can be learned by

(5)         θ←arg minθ∑(i,j)∈Sζij(θ)Mij(θ)←θ−β∇θℒmeta(Dit,Djt)

where *M*_*ij*_:θ → θ_*ij*_ is a function which maps the model initialization θ into the optimal task-specific parameter θ_*ij*_. Similar to the model-agnostic meta-learning (MAML) (Finn et al., 2017), *M*_*ij*_(θ) can be instantiated by one or a few gradient descent updates in practice. In this case, the meta-training loss is given by $L^{meta}\left(D_{i}^{t},D_{j}^{t}\right)={\hat{\epsilon }}_{i}^{t}\left(M_{ij}\left(\theta \right)\right)+\eta \cdot {\hat{d}}_{H\Delta H}\left(D_{i}^{t},D_{j}^{t};M_{ij}\left(\theta \right)\right)$ over the training set.
As illustrated in Algorithm 1, the predictive function is incrementally learned for the target task at every historical time stamp, and then the pseudo-labels of unlabeled target examples can be inferred.

- **Meta-Testing:** The optimal parameters θ_*N*+1_ on the newest target task $D_{N+1}^{t}$ could be learned by fine-tuning the optimal model initialization θ on a selective meta-pair of tasks $\left(D_{k}^{t},D_{N+1}^{t}\right)$.

(6) $$\begin{array}{l} \theta_{N+1}=M_{k\left(N+1\right)}\left(\theta \right)\leftarrow \theta -\beta \nabla_{\theta }L^{meta}\left(D_{k}^{t},D_{N+1}^{t}\right) \end{array}$$

where θ is the optimized model initialization learned in the meta-training phase. Here we choose the meta-pair of tasks $\left(D_{k}^{t},D_{N+1}^{t}\right)$ by estimating the sampling probability *w*_*k*(*N*+1)_ (*k* = 0, 1, ⋯ , *N*) and choosing *k* with the largest value *w*_*k*(*N*+1)_.

**Algorithm 1:**
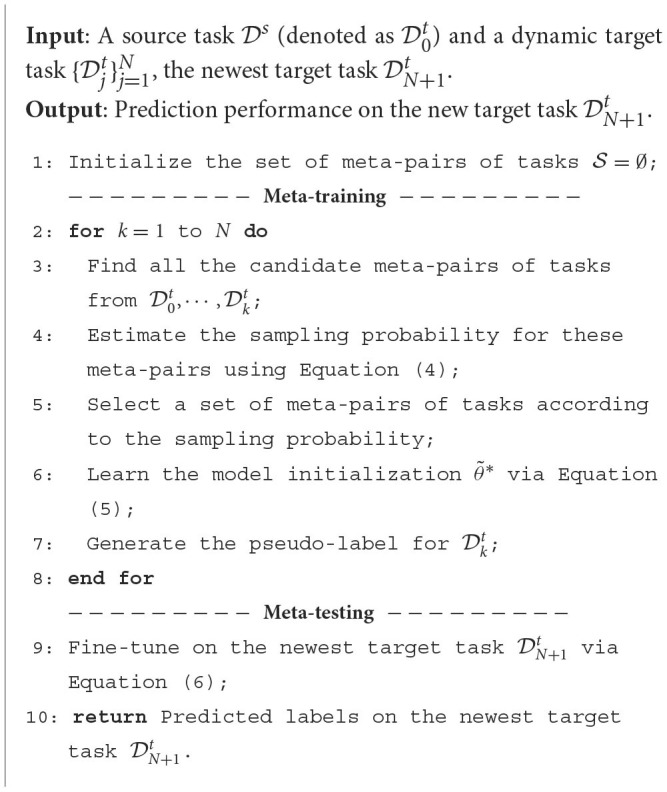
Learning to Schedule (L2S).

## 6. Experiments

In this section, we provide the empirical analysis of L2S framework on various data sets.

### 6.1. Experimental setup

We used the following publicly available image data sets:

- Rotating MNIST (Kumar et al., 2020): The original MNIST (LeCun et al., 1998) is a digital image data set with 60,000 images from 10 categories. Rotating MNIST is a semi-synthetic version of MNIST where each image is rotated by a degree. Following Bobu et al. (2018) and Kumar et al. (2020), we rotate each image by an angle for generating the time-evolving classification task. More specifically, for the source task, we randomly choose 32 images and then rotate them by an angle between 0 and 10 degrees. All the images in the source task are associated with class labels. For the time-evolving target task, we randomly choose 32 images at every time stamp *j* (*j* = 1, ⋯ , 35) and rotate them by an angle between 10·*j* and 10·(*j*+1) degrees. It can be seen that in this case, the data distribution of the target task is continuously evolving over time. Therefore, we denote the aforementioned Rotating MNIST as a data set “with continuous evolvement.” In contrast, we consider the dynamic transfer learning scenarios “with large distribution shift,” where the samples at the last 18 time stamps of the target task are randomly shuffled. That is, the target task might not be evolving smoothly with respect to the rotation degree.
- ImageCLEF-DA (Long et al., 2017): ImageCLEF-DA has three image classification tasks: Caltech-256 (C), ImageNet ILSVRC 2012 (I) and Pascal VOC 2012 (P). Following Wu and He (2022b), we generate the time evolving target task by adding random noise and rotation to the original images. For example, if we consider Caltech-256 (C) as the target task, we can generate a time-evolving target task by rotating the original images of Caltech-256 with a degree *O*_*d*_(*j*) (*j* = 1, 2⋯ , 5 is the time stamp) and adding the random salt&pepper noise with the magnitude *O*_*n*_(*j*), i.e., *O*_*d*_(*j*) = 15·(*j*−1), *O*_*n*_(*j*) = 0.01·(*j*−1), *N* = 4.

Following Bobu et al. (2018) and Wu and He (2022b), we report both the classification accuracy on the newest target task (Acc) and the average classification accuracy on the historical target tasks (H-Acc) in the experiments. The comparison baselines we used in the experiments include: (1) static transfer learning approaches: SourceOnly, DAN (Long et al., 2015), DANN (Ganin et al., 2016), and MDD (Zhang et al., 2019); and (2) dynamic transfer learning: CUA (Bobu et al., 2018), GST (Kumar et al., 2020), L2E (Wu and He, 2022b), and our proposed L2S framework. For a fair comparison, all the methods use the same base models for feature extraction, e.g., LeNet for Rotating MNIST and ResNet-18 (He et al., 2016) for ImageCLEF-DA. In addition, we set η = 1, β = 0.01 and the number of inner epochs in *M*_*ij*_(θ) as 1. All the experiments are performed on a Windows machine with four 3.80GHz Intel Cores, 64GB RAM and two NVIDIA Quadro RTX 5000 GPUs.

### 6.2. Results

**Figure 3:**
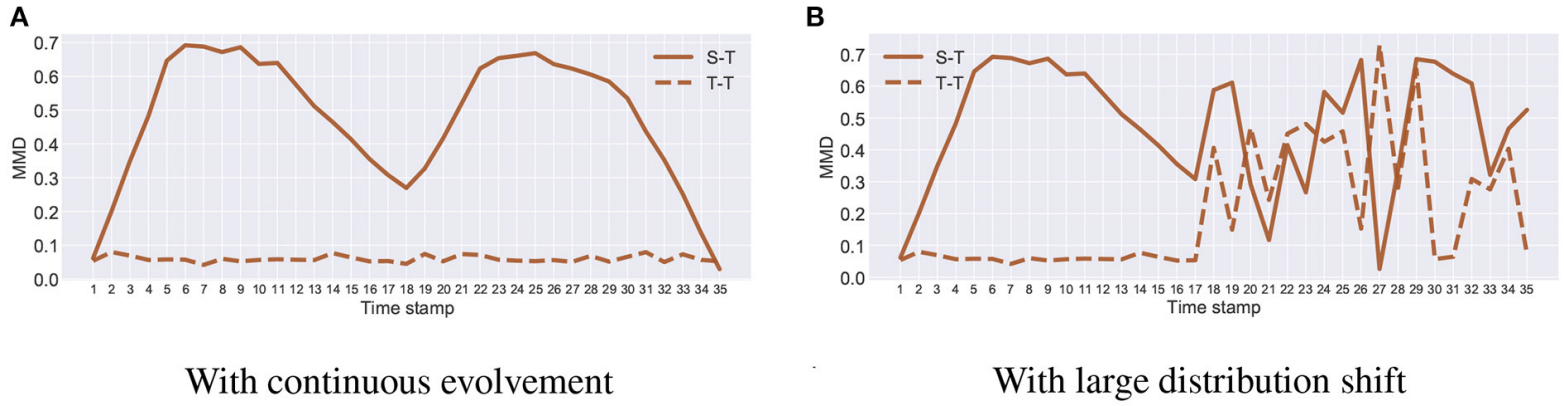
Rotating MNIST with **(A)** continuous evolvement and **(B)** large distribution shift.

**Figure 4:**
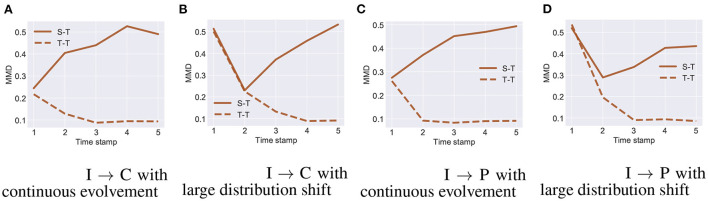
I → C on ImageCLEF-DA with **(A)** continuous evolvement, **(B)** large distribution shift. I → P on ImageCLEF-DA with **(C)** continuous evolvement, **(D)** large distribution shift.

Figures 3, 4 show the distribution shift in the dynamic transfer learning tasks, where “S-T" denotes the distribution difference $d\left(D^{s},D_{j}^{t}\right)$ between the source and the target at every time stamp and “T-T" denotes the distribution difference $d\left(D_{j-1}^{t},D_{j}^{t}\right)$ of the target at consecutive time stamp. Here we use maximum mean discrepancy (MMD) (Gretton et al., 2012) to measure the distribution difference across tasks. We see that when the target task is continuously evolving over time, $d\left(D_{j-1}^{t},D_{j}^{t}\right)$ is small. This enables gradual knowledge transferability in the target task. If there exists a large distribution shift at some times, i.e., $d\left(D_{j-1}^{t},D_{j}^{t}\right)$ is large, the strategy of gradual knowledge transferability might fail. In Figures 3, 4, the large distribution shift happened in the time stamps 17–35 on Rotating MNIST and time stamp 1 on I → C/P.

Tables 1, 2 provides the experimental results of L2S as well as baselines on Rotating MNIST and Image-CLEF data sets. We have the following observations from the results. On the one hand, when the target task is continuously evolving over time, most dynamic transfer learning baselines can achieve satisfactory performance on both the newest and historical target tasks. The baseline GST (Kumar et al., 2020) fails on Rotating MNIST, because the self-training approach might be more likely to accumulate the classification error when the target task is evolving for a long time. On the other hand, the performance of CUA (Bobu et al., 2018) and L2E (Wu and He, 2022b) drops significantly when there is a large distribution shift within the target task at some time stamp. In contrast, by adaptively selecting the meta-pairs of tasks, the proposed L2S framework can mitigate the issue of the potential large distribution shift in the targe task. Specifically, compared to L2E (Wu and He, 2022b), L2S improves the performance by a large margin. This confirms the efficacy of the proposed progressive meta-pair scheduler.

**Table 1:** Results of dynamic transfer learning on Rotating MNIST.

**Methods**	**With continuous evolvement**		**With large distribution shift**	
	**Acc**	**H-Acc**	**Acc**	**H-Acc**
SourceOnly	1.0000	0.4393	0.3437	0.4393
DAN (Long et al., 2015)	1.0000	0.4518	0.5625	0.4830
DANN (Ganin et al., 2016)	1.0000	0.3884	0.3750	0.4000
MDD (Zhang et al., 2019)	1.0000	0.4250	0.4063	0.4482
CUA (Bobu et al., 2018)	0.9375	0.9277	0.4375	0.8259
GST (Kumar et al., 2020)	0.0625	0.1062	0.1250	0.2259
L2E (Wu and He, 2022b)	0.9688	0.9795	0.6250	0.7179
L2S	**1.0000**	**0.9991**	**0.9687**	**0.9116**

The best results are indicated in bold.

**Table 2:** Results of dynamic transfer learning on ImageCLEF-DA.

**Methods**	**With continuous evolvement**				**With large distribution shift**			

	**I**→**C**		**I**→**P**		**I**→**C**		**I**→**P**	
	**Acc**	**H-Acc**	**Acc**	**H-Acc**	**Acc**	**H-Acc**	**Acc**	**H-Acc**
SourceOnly	0.3125	0.4250	0.2812	0.3938	0.3125	0.4125	0.2187	0.2562
DAN (Long et al., 2015)	0.2500	0.4000	0.2187	0.2688	0.3750	0.3750	0.2500	0.2625
DANN (Ganin et al., 2016)	0.3125	0.4438	0.3125	0.4188	0.3125	0.4125	0.1875	0.2750
MDD (Zhang et al., 2019)	0.3437	0.4750	0.3125	0.4562	0.3125	0.4062	0.2500	0.3188
CUA (Bobu et al., 2018)	0.4063	0.5125	0.5312	0.5438	0.4375	0.4625	0.3437	0.4000
GST (Kumar et al., 2020)	0.5000	0.5312	0.4375	0.4312	0.2812	0.3062	0.2500	0.2562
L2E (Wu and He, 2022b)	**0.5625**	**0.6875**	0.5625	0.5875	0.3750	0.4812	0.3750	**0.4812**
L2S	**0.5625**	0.6125	**0.6562**	**0.6188**	**0.4375**	**0.5500**	**0.4375**	**0.4812**

The best results are indicated in bold.

### 6.3. Analysis

**Figure 5:**
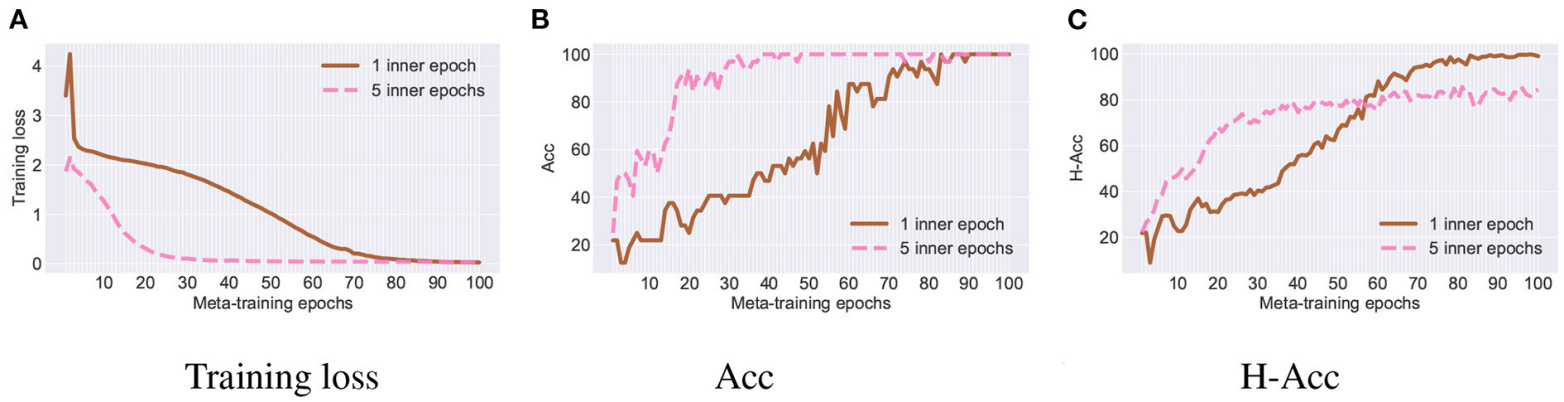
Ablation study with different number of inner epochs. **(A)** Training loss. **(B)** Acc. **(C)** H-Acc.

We provide the ablation study of our L2S framework with respect to the number of inner training epochs. The results on the newest target task of Rotating MNIST are shown in Figure 5, where we use 1 or 5 inner epochs for our meta-learning framework. We see that using more inner epochs can improve the convergence of L2S but it sacrifices the classification accuracy on the historical target task. This is because L2S with more inner epochs would enforce the fine-tuned model to be more task-specific. Thus, we set the number of inner epochs as 1 in our experiments.

## 7. Conclusion

In this paper, we study the problem of dynamic transfer learning from a labeled source task to an unlabeled dynamic target task. We start by deriving the generalization error bounds of dynamic transfer learning by assigning the meta-pairs of tasks with different weights. This allows us to provide the tighter error bound when there is a large distribution shift of the target task at some time stamp. Then we develop a novel meta-learning framework L2S with progressive meta-task scheduler for dynamic transfer learning. Extensive experiments on several image data sets demonstrate the effectiveness of the proposed L2S framework over state-of-the-art baselines.

## Data availability statement

The original contributions presented in the study are included in the article/Supplementary material, further inquiries can be directed to the corresponding author.

## Author contributions

JW and JH work together to develop a new theoretical understanding and algorithms for dynamic transfer learning. Both authors contributed to the article and approved the submitted version.

## Funding

This work is supported by the National Science Foundation under Award Nos. IIS-1947203, IIS-2117902, and IIS-2137468 and Agriculture and Food Research Initiative (AFRI) Grant No. 2020-67021-32799/project accession no. 1024178 from the USDA National Institute of Food and Agriculture.

## Conflict of interest

The authors declare that the research was conducted in the absence of any commercial or financial relationships that could be construed as a potential conflict of interest.

## Publisher's note

All claims expressed in this article are solely those of the authors and do not necessarily represent those of their affiliated organizations, or those of the publisher, the editors and the reviewers. Any product that may be evaluated in this article, or claim that may be made by its manufacturer, is not guaranteed or endorsed by the publisher.

## Author disclaimer

The views and conclusions are those of the authors and should not be interpreted as representing the official policies of the funding agencies or the government.

## Footnotes

^1^It can also be generalized to the scenarios (Wu and He, 2022b) where the knowledge is transferred from a dynamic source task to a dynamic target task.

^2^Here we assume that it generates the same number of examples at every time stamp, i.e., $m^{s}=m_{1}^{t}=\cdots =m_{N+1}^{t}=m$, but the theoretical results can also be generalized into the scenarios with different number of samples in source and target tasks.
