# Supplementary material for: Dynamic transfer learning with progressive meta-task scheduler
Source: Front Big Data. 2022 Nov 3;5:1052972. doi: 10.3389/fdata.2022.1052972 (PMC9669596; doi:10.3389/fdata.2022.1052972)
Supplement: Supplementary file 1 [file Data_Sheet_1.PDF]

Supplementary Material

1 THEORETICAL RESULTS

In this section, we show the detailed proof for all the theorems and corollaries involved in the paper.

1.1 Proof of Theorem 4.1

Theorem 4.1 states that let $\mathcal{H}$ be a hypothesis space of VC dimension d. If there are m labeled source examples i.i.d. drawn from $\mathcal{D}^s$ (denoted as $\mathcal{D}_0^t$ as well) and m unlabeled target examples i.i.d. drawn from $\mathcal{D}_j^t$ for each time stamp $j = 1, \cdots, N+1$, then for any $\delta > 0$ and $h \in \mathcal{H}$, with probability at least $1-\delta$, the expected error of the newest target task $\mathcal{D}_{N+1}^t$ can be bounded in the following.

$$\epsilon_{N+1}^t(h) \leq \sum_{i=0}^{N} \sum_{j=i+1}^{N+1} w_{ij} \left(\hat{\epsilon}_i^t(h) + \eta_{ij} \cdot \hat{d}_{\mathcal{H}\Delta\mathcal{H}} \left(\mathcal{D}_i^t, \mathcal{D}_j^t \right) \right)$$
$$+ \mathcal{O}\left(\lambda + \sqrt{\frac{d \log(2m) + \log(2/\delta) + \sum_{i=0}^{N} \sum_{j=i+1}^{N+1} w_{ij}^2 \log(1/\delta)}{2m}} \right)$$

where $\sum_{i=0}^{N} \sum_{j=i+1}^{N+1} w_{ij} = 1$, and $w_{ij} \geq 0$ if $i < j$, $w_{ij} = 0$ otherwise. $\eta_{ij} = \frac{1}{2}$ if $1 \leq j \leq N$ and $i < j$, and $\eta_{ij} = \frac{1}{2}\left(1 + \frac{\sum_{k=0}^{i-1} w_{ki}}{w_{ij}}\right)$ if $j = N+1$ and $i < j$, $\eta_{ij} = 0$ otherwise. Here λ denotes the combined error of the ideal hypothesis over all the tasks, i.e., $\lambda = \min_{h\in\mathcal{H}} \sum_{i=0}^{N+1} \epsilon_i^t(h)$, and $\hat{d}_{\mathcal{H}\Delta\mathcal{H}}(\cdot,\cdot)$ denotes the empirical estimate of $\mathcal{H}$-divergence over finite examples.

PROOF. Following (Ben-David et al., 2010) and (Mohri and Muñoz Medina, 2012), we show our theorem as follows. We first define a function g over sample set $\mathcal{B} = \left\{ \{(\mathbf{x}_{i0}, y_{i0})\}_{i=1}^m, \cdots, \{(\mathbf{x}_{iN}, y_{iN})\}_{i=1}^m \right\}$.

$$g(\mathcal{B}) = \sup_{h\in\mathcal{H}} \epsilon_{N+1}^t(h) - \sum_{i=0}^{N} \sum_{j=i+1}^{N+1} w_{ij} \hat{\epsilon}_i^t(h) = \sup_{h\in\mathcal{H}} \sum_{i=0}^{N} \sum_{j=i+1}^{N+1} w_{ij} \left(\epsilon_{N+1}^t(h) - \hat{\epsilon}_i^t(h) \right)$$

Let $\mathcal{B}$ and $\mathcal{B}'$ be two sample sets containing only one different sample for estimating $w_{ij}\hat{\epsilon}_i^t(h) = \frac{w_{ij}}{m} \sum_{k=1}^m \mathcal{L}\left(h\left(\mathbf{x}_{im}^t\right), y_{im}^t \right)$. Then we have

$$\left| g(\mathcal{B}) - g(\mathcal{B}') \right| = \frac{1}{m} \sup_{h\in\mathcal{H}} \left| \mathcal{L}\left(h\left(\mathbf{x}\right), y\right) - \mathcal{L}\left(h\left(\mathbf{x}'\right), y'\right) \right| \leq \frac{w_{ij}}{m}$$

Thus, based on McDiarmid's inequality, the following holds:

$$\Pr\left[g(\mathcal{B}) - \mathbb{E}_{\mathcal{B}}\left[g(\mathcal{B}) \right] \geq \epsilon \right] \leq \exp\left(- \frac{2m\epsilon^2}{\sum_{i=0}^{N} \sum_{j=i+1}^{N+1} w_{ij}^2} \right)$$

Based on Hoeffding's inequality, we have

$$\Pr\left[\left|\epsilon_i^t(h) - \hat{\epsilon}_i^t(h)\right| \geq \epsilon\right] \leq 2\exp\left(-\frac{2m\epsilon^2}{M^2}\right)$$

Then we have the following result.

$$\begin{aligned}
\mathbb{E}_{\mathcal{B}}\left[g(\mathcal{B})\right] &= \mathbb{E}_{\mathcal{B}}\left[\sup_{h\in\mathcal{H}}\sum_{i=0}^{N}\sum_{j=i+1}^{N+1} w_{ij}\left(\epsilon_{N+1}^t(h) - \hat{\epsilon}_i^t(h)\right)\right] \\
&= \mathbb{E}_{\mathcal{B}}\left[\sup_{h\in\mathcal{H}}\sum_{i=0}^{N}\sum_{j=i+1}^{N+1} w_{ij}\left(\epsilon_{N+1}^t(h) - \epsilon_j^t(h) + \epsilon_j^t(h) - \epsilon_i^t(h) + \epsilon_i^t(h) - \hat{\epsilon}_i^t(h)\right)\right] \\
&\leq \mathbb{E}_{\mathcal{B}}\left[\sup_{h\in\mathcal{H}}\sum_{i=0}^{N}\sum_{j=i+1}^{N+1} w_{ij}\left(\epsilon_j^t(h) - \epsilon_i^t(h)\right)\right] + \mathbb{E}_{\mathcal{B}}\left[\sup_{h\in\mathcal{H}}\sum_{i=0}^{N}\sum_{j=i+1}^{N+1} w_{ij}\left(\epsilon_{N+1}^t(h) - \epsilon_j^t(h)\right)\right] \\
&\quad + \mathbb{E}_{\mathcal{B}}\left[\sup_{h\in\mathcal{H}}\sum_{i=0}^{N}\sum_{j=i+1}^{N+1} w_{ij}\left(\epsilon_i^t(h) - \hat{\epsilon}_i^t(h)\right)\right] \\
&\leq \sum_{i=0}^{N}\sum_{j=i+1}^{N+1} w_{ij}\left(\frac{1}{2}d_{\mathcal{H}\Delta\mathcal{H}}\left(\mathcal{D}_i^t, \mathcal{D}_j^t\right) + \lambda_{ij}\right) + \sum_{i=0}^{N}\sum_{j=i+1}^{N+1} w_{ij}\left(\frac{1}{2}d_{\mathcal{H}\Delta\mathcal{H}}\left(\mathcal{D}_j^t, \mathcal{D}_{N+1}^t\right) + \lambda_{j(N+1)}\right) \\
&\quad + \sum_{i=0}^{N}\sum_{j=i+1}^{N+1} w_{ij}\left(\sqrt{\frac{\log(2/\delta)}{2m}}\right) \\
&= \sum_{i=0}^{N}\sum_{j=i+1}^{N+1} w_{ij}\left(\frac{1}{2}d_{\mathcal{H}\Delta\mathcal{H}}\left(\mathcal{D}_i^t, \mathcal{D}_j^t\right) + \frac{1}{2}d_{\mathcal{H}\Delta\mathcal{H}}\left(\mathcal{D}_j^t, \mathcal{D}_{N+1}^t\right) + \lambda_{ij} + \lambda_{j(N+1)}\right) + \sqrt{\frac{\log(2/\delta)}{2m}} \\
&\leq \sum_{i=0}^{N}\sum_{j=i+1}^{N+1} w_{ij}\left(\frac{1}{2}d_{\mathcal{H}\Delta\mathcal{H}}\left(\mathcal{D}_i^t, \mathcal{D}_j^t\right) + \frac{1}{2}d_{\mathcal{H}\Delta\mathcal{H}}\left(\mathcal{D}_j^t, \mathcal{D}_{N+1}^t\right) + 2\lambda\right) + \sqrt{\frac{\log(2/\delta)}{2m}} \\
&\leq \sum_{i=0}^{N}\sum_{j=i+1}^{N+1} w_{ij}\eta_{ij}d_{\mathcal{H}\Delta\mathcal{H}}\left(\mathcal{D}_i^t, \mathcal{D}_j^t\right) + 2\lambda + \sqrt{\frac{\log(2/\delta)}{2m}} \\
&\leq \sum_{i=0}^{N}\sum_{j=i+1}^{N+1} w_{ij}\eta_{ij}\left(\hat{d}_{\mathcal{H}\Delta\mathcal{H}}\left(\mathcal{D}_i^t, \mathcal{D}_j^t\right) + 4\sqrt{\frac{d\log(2m) + \log(2/\delta)}{m}}\right) + 2\lambda + \sqrt{\frac{\log(2/\delta)}{2m}} \\
&= \sum_{i=0}^{N}\sum_{j=i+1}^{N+1} w_{ij}\eta_{ij}\hat{d}_{\mathcal{H}\Delta\mathcal{H}}\left(\mathcal{D}_i^t, \mathcal{D}_j^t\right) + \mathcal{O}\left(\lambda + \sqrt{\frac{d\log(2m) + \log(2/\delta)}{m}}\right)
\end{aligned}$$

where

$$\eta_{ij} = \begin{cases} \frac{1}{2}, & \text{if } 1 \leq j \leq N \\ \frac{1}{2}\left(1 + \frac{\sum_{k=0}^{i-1} w_{ki}}{w_{ij}}\right), & \text{if } j = N+1 \end{cases}$$

Therefore,

$$g(\mathcal{B}) = \sup_{h\in\mathcal{H}} \epsilon^t_{N+1}(h) - \sum_{i=0}^{N}\sum_{j=i+1}^{N+1} w_{ij}\hat{\epsilon}^t_i(h)$$

$$\leq \mathbb{E}_{\mathcal{B}}\left[g(\mathcal{B})\right] + \sqrt{\frac{\sum_{i=0}^{N}\sum_{j=i+1}^{N+1} w_{ij}^2 \log(1/\delta)}{2m}}$$

$$\leq \sum_{i=0}^{N}\sum_{j=i+1}^{N+1} w_{ij}\eta_{ij}\hat{d}_{\mathcal{H}\Delta\mathcal{H}}\left(\mathcal{D}^t_i, \mathcal{D}^t_j\right) + \mathcal{O}\left(\lambda + \sqrt{\frac{d\log(2m) + \log(2/\delta) + \sum_{i=0}^{N}\sum_{j=i+1}^{N+1} w_{ij}^2\log(1/\delta)}{2m}}\right)$$

which completes the proof.

1.2 Proof of Theorem 4.2

Theorem 4.2 states that let $\mathcal{H}$ be a hypothesis space of VC dimension d. If there are m labeled source examples i.i.d. drawn from $\mathcal{D}^s$ (denoted as $\mathcal{D}^t_0$ as well) and m unlabeled target examples i.i.d. drawn from $\mathcal{D}^t_j$ for each time stamp $j = 1, \cdots, N+1$, then for any $\delta > 0$ and a proper inner learning rate β, with probability at least $1-\delta$, the expected error of the newest target task $\mathcal{D}^t_{N+1}$ can be bounded in the following.

$$\epsilon^t_{N+1}(h_{N+1}) \leq \sum_{i=0}^{N}\sum_{j=i+1}^{N+1} w_{ij}\Big(\hat{\epsilon}^t_i(h_i) + \eta_{ij}\cdot \hat{d}_{\mathcal{H}\Delta\mathcal{H}}\left(\mathcal{D}^t_i, \mathcal{D}^t_j\right)\Big)$$

$$+\,\mathcal{O}\left(\sum_{i=0}^{N}\left(\frac{1}{m}\sum_{k=1}^{m}\left|\left|\nabla_\theta \bar{h}(\mathbf{x}_{ki})\right|\right|\right)^2 + \lambda + \sqrt{\frac{d\log(2m) + \log(2/\delta) + \sum_{i=0}^{N}\sum_{j=i+1}^{N+1} w_{ij}^2\log(1/\delta)}{m}}\right)$$

where $\sum_{i=0}^{N}\sum_{j=i+1}^{N+1} w_{ij} = 1$, and $w_{ij} \geq 0$ if $i<j$, $w_{ij} = 0$ otherwise. $\eta_{ij} = \frac{1}{2}$ if $1 \leq j \leq N$ and $i < j$, and $\eta_{ij} = \frac{1}{2}\left(1 + \frac{\sum_{k=0}^{i-1} w_{ki}}{w_{ij}}\right)$ if $j = N+1$ and $i<j$, $\eta_{ij} = 0$ otherwise. Here λ denotes the combined error of the ideal hypothesis over all the tasks, i.e., $\lambda = \min_{h\in\mathcal{H}}\sum_{i=0}^{N+1}\epsilon^t_i(h)$, and $\hat{d}_{\mathcal{H}\Delta\mathcal{H}}(\cdot,\cdot)$ denotes the empirical estimate of $\mathcal{H}$-divergence over finite examples.

PROOF. With a proper learning rate β, we have $\epsilon^t_{N+1}(h_{N+1}) \leq \epsilon^t_{N+1}(\bar{h})$, because h_{N+1} is the updated hypothesis of $\bar{h}$ with one step gradient descent. Using Theorem 4.1, the following holds

$$\epsilon^t_{N+1}(\bar{h}) \leq \sum_{i=0}^{N}\sum_{j=i+1}^{N+1} w_{ij}\Big(\hat{\epsilon}^t_i(\bar{h}) + \eta_{ij}\cdot\hat{d}_{\mathcal{H}\Delta\mathcal{H}}\left(\mathcal{D}^t_i, \mathcal{D}^t_j\right)\Big)$$

$$+\,\mathcal{O}\left(\lambda + \sqrt{\frac{d\log(2m) + \log(2/\delta) + \sum_{i=0}^{N}\sum_{j=i+1}^{N+1} w_{ij}^2\log(1/\delta)}{2m}}\right)$$

Moreover, we can represent the outputs of the hypothesis $\bar{h}(\mathbf{x})$ by their first order Taylor expansion,

$$h_i(\mathbf{x}) - \bar{h}(\mathbf{x}) \approx \nabla_\theta \bar{h}(\mathbf{x}) \left(-\beta \frac{1}{m} \sum_{k=1}^{m} \nabla_\theta \mathcal{L}(\bar{h}(\mathbf{x}_{ki}), y_{ki}) \right)$$

Then

$$\begin{aligned}
\hat{\epsilon}_i^t(\bar{h}) &= \hat{\epsilon}_i^t(h_i) + \hat{\epsilon}_i^t(\bar{h}) - \hat{\epsilon}_i^t(h_i) \\
&\le \hat{\epsilon}_i^t(h_i) + \frac{1}{m} \sum_{k=1}^{m} \mathcal{L}(\bar{h}(\mathbf{x}_{ki}), y_{ki}) - \frac{1}{m} \sum_{k=1}^{m} \mathcal{L}(h_i(\mathbf{x}_{ki}), y_{ki}) \\
&\le \hat{\epsilon}_i^t(h_i) + \frac{1}{m} \sum_{k=1}^{m} \left| \bar{h}(\mathbf{x}_{ki}) - h_i(\mathbf{x}_{ki}) \right| \\
&\le \hat{\epsilon}_i^t(h_i) + \frac{1}{m} \sum_{k=1}^{m} \left| \nabla_\theta \bar{h}(\mathbf{x}_{ki}) \left(\beta \frac{1}{m} \sum_{k=1}^{m} \nabla_\theta \mathcal{L}(\bar{h}(\mathbf{x}_{ki}), y_{ki}) \right) \right| \\
&= \hat{\epsilon}_i^t(h_i) + \frac{1}{m} \sum_{k=1}^{m} \left| \nabla_\theta \bar{h}(\mathbf{x}_{ki}) \left(\beta \frac{1}{m} \sum_{k=1}^{m} \text{sign}\left(\bar{h}(\mathbf{x}_{ki}) - y_{ki} \right) \cdot \nabla_\theta \bar{h}(\mathbf{x}_{ki}) \right) \right| \\
&\le \hat{\epsilon}_i^t(h_i) + \frac{\beta}{m^2} \sum_{k=1}^{m} \left|\left| \nabla_\theta \bar{h}(\mathbf{x}_{ki}) \right|\right| \cdot \left|\left| \sum_{k=1}^{m} \text{sign}\left(\bar{h}(\mathbf{x}_{ki}) - y_{ki} \right) \cdot \nabla_\theta \bar{h}(\mathbf{x}_{ki}) \right|\right| \\
&\le \hat{\epsilon}_i^t(h_i) + \frac{\beta}{m^2} \sum_{k=1}^{m} \left|\left| \nabla_\theta \bar{h}(\mathbf{x}_{ki}) \right|\right| \cdot \sum_{k=1}^{m} \left|\left| \text{sign}\left(\bar{h}(\mathbf{x}_{ki}) - y_{ki} \right) \cdot \nabla_\theta \bar{h}(\mathbf{x}_{ki}) \right|\right| \\
&\le \hat{\epsilon}_i^t(h_i) + \beta \left(\frac{1}{m} \sum_{k=1}^{m} \left|\left| \nabla_\theta \bar{h}(\mathbf{x}_{ki}) \right|\right| \right)^2
\end{aligned}$$

where we use the following Cauchy–Schwarz inequality

$$\left| \nabla_\theta \bar{h}(\mathbf{x}_{ki}) \left(\sum_{k=1}^{m} \text{sign}\left(\bar{h}(\mathbf{x}_{ki}) - y_{ki} \right) \cdot \nabla_\theta \bar{h}(\mathbf{x}_{ki}) \right) \right| \le \left|\left| \nabla_\theta \bar{h}(\mathbf{x}_{ki}) \right|\right| \left|\left| \sum_{k=1}^{m} \text{sign}\left(\bar{h}(\mathbf{x}_{ki}) - y_{ki} \right) \cdot \nabla_\theta \bar{h}(\mathbf{x}_{ki}) \right|\right|$$

This completes the proof.

1.3 Proof of Corollary 4.3

Corollary 4.3 states that with the same assumptions in Theorem 4.1, for any $\delta > 0$ and $h \in \mathcal{H}$, there exist $w_{ij} \ge 0$ and $\eta_{ij} \ge 0$, with probability at least $1 - \delta$, the expected error of the newest target task $\mathcal{D}_{N+1}^t$ can be bounded in the following.

$$\epsilon_{N+1}^t(h) \le \sum_{i=0}^{N} \sum_{j=i+1}^{N+1} w_{ij} \left(\hat{\epsilon}_i^t(h) + \eta_{ij} \cdot \hat{d}\left(\mathcal{D}_i^t, \mathcal{D}_j^t \right) \right) + \Omega \tag{S1}$$

where $\hat{d}(\cdot, \cdot)$ can be instantiated with existing distribution discrepancy measures, including discrepancy distance (Mansour et al., 2009), maximum mean discrepancy (Long et al., 2015), Wasserstein distance (Shen et al., 2018), f-divergence (Acuna et al., 2021), etc. Here Ω denotes the corresponding sample complexity when the distribution discrepancy measure is selected.

PROOF. This corollary can be proved by extending the generalization results with different discrepancy measures from static to dynamic transfer learning setting, using similar idea as Theorem 4.1.

REFERENCES

Acuna, D., Zhang, G., Law, M. T., and Fidler, S. (2021). f-domain adversarial learning: Theory and algorithms. In *International Conference on Machine Learning* (PMLR), 66–75

Ben-David, S., Blitzer, J., Crammer, K., Kulesza, A., Pereira, F., and Vaughan, J. W. (2010). A theory of learning from different domains. *Machine learning* 79, 151–175

Long, M., Cao, Y., Wang, J., and Jordan, M. (2015). Learning transferable features with deep adaptation networks. In *International conference on machine learning* (PMLR), 97–105

Mansour, Y., Mohri, M., and Rostamizadeh, A. (2009). Domain adaptation: Learning bounds and algorithms. In *22nd Conference on Learning Theory, COLT 2009*

Mohri, M. and Muñoz Medina, A. (2012). New analysis and algorithm for learning with drifting distributions. In *International Conference on Algorithmic Learning Theory* (Springer), 124–138

Shen, J., Qu, Y., Zhang, W., and Yu, Y. (2018). Wasserstein distance guided representation learning for domain adaptation. In *Proceedings of the AAAI Conference on Artificial Intelligence*. vol. 32
